# Life Cycle and Biometric Study of *Hydrotaea capensis* (Wiedemann, 1818) (Diptera, Muscidae), a Species of Forensic Interest

**DOI:** 10.3390/insects13060531

**Published:** 2022-06-09

**Authors:** María Pérez-Marcos, Mª Dolores García, Elena López-Gallego, Mª José Ramírez-Soria, Mª Isabel Arnaldos

**Affiliations:** 1 Biological Control and Ecosystem Services Laboratory, Crop Protection Department, Institute of Agricultural and Environmental Research and Development of Murcia (IMIDA), C/Mayor s/n, E-30150 Murcia, Spain; elena.lopez5@carm.es (E.L.-G.); mjramirezsoria@gmail.com (M.J.R.-S.); 2 Department of Zoology, Faculty of Biology, Campus of International Excellence Mare Nostrum, University of Murcia, 30100 Murcia, Spain; mdgarcia@um.es (M.D.G.); miarnald@um.es (M.I.A.); 3 External Service of Forensic Sciences and Techniques, University of Murcia, 30100 Murcia, Spain

**Keywords:** biometric characteristics, developmental rates, forensic entomology, life cycle, temperature effects

## Abstract

**Simple Summary:**

From the point of view of forensic entomology, the study of the development times of the species and the factors conditioning them are issues of great importance to estimate the minPMI. *Hydrotaea capensis* is a Muscidae (Diptera) of forensic interest because of its colonization preferences. Thus, there is a need to have more precise data about its biology to gain insights into the interpretation of its presence in corpses. In this study, the *H. capensis* life cycle was studied at four constant temperatures, 18, 20, 25 and 30 °C, by recording the duration of its different developmental stages and the length reached in each larval stage, as well as some biometric characteristics of the emerged adults. Our work is the first to provide data on the duration of the life cycle of this species and pointed to some alar features as relevant biometric adult indicators to be considered.

**Abstract:**

One of the most important and perhaps most used applications of forensic entomology concerns the estimation of the minimum post-mortem interval (minPMI), defined as the time interval between death and the actual finding of a corpse. Some Diptera species are critical in these studies because they are the first ones capable of detecting and finding a corpse and are selectively attracted by its decomposing status. Thus, the knowledge of the micromorphology of their preimaginal stages and of their life cycles within a time frame constitutes solid indicators for estimating the minPMI. *Hydrotaea capensis* is a Muscidae of forensic interest usually considered as a late colonizer of corpses. It is widely distributed, living mainly in warm regions, and present in a wide variety of habitats. In this study, the *H. capensis* life cycle was studied at four constant temperatures, 18°, 20°, 25° and 30 °C, by recording the duration of its different developmental stages, including the length reached in each larval stage, as well as some biometric characteristics of the emerged adults. Significant differences were observed in the average time of development of most larval stages, with a longer duration at low temperatures, and in the length of each larval stage depending on the temperature, but, in this case, without a clear pattern. Moreover, significant differences were found in some alar features, pointing to them as a relevant indicator to be considered. The data provided will assist forensic entomologists to make more accurate minPMI estimations in cases where *H. capensis* is present.

## 1. Introduction

Insects detect the presence of a corpse at great distances, colonizing it rapidly and becoming the first to utilize this resource [1]. They are considered among the most powerful tools for determining the period between death and the time of finding a corpse, or the minimum postmortem interval (minPMI) [2,3,4]. The characteristics of the life cycles or the growth curves of the larval bodies are some of the entomological indicators currently accepted to estimate the minPMI [5,6,7,8]. However, these processes are affected by numerous external factors, mainly climatological. Since insects are poikilothermic, it is known that temperature can be one of the most critical factors because of its relation to the insect developmental features specific to each species and, therefore, defining a specific threshold below which colonization and development processes cannot start [5,8,9,10]. The interval preceding the appearance of a given species on a corpse is named the pre-appearance interval (PAI) or the pre-colonization interval, the primary colonizers being very useful to estimate the more accurate minPMI [10]. However, in the case of middle- and late-arriving insects, the estimation is less accurate because they provide a wider range of minPMI, which is probably the reason why they are used less for minPMI estimation [6]. Thus, further investigation of the relationships of late colonizing species with environmental factors, in particular with temperature, and their colonization times, will allow the applications of entomology in forensic sciences to be increasingly precise.

The majority of the invertebrate fauna founded on corpses are Diptera, which are considered primary colonizers as they include the first species capable of detecting and finding a corpse, even from great distances, and are selectively attracted by the decomposing status of the body [11,12,13]. This order includes numerous families, Calliphoridae, Muscidae and Sarcophagidae being the most interesting from a forensic point of view and, usually, favorably considered for minPMI estimation since some of their life cycles are generally well-known [14,15,16,17]. Insects usually colonize corpses in a successional process parallel to the decomposition of the different bodies. The first colonizing species usually belong to the family Calliphoridae; however, there is no single established classification based on a single colonization sequence and it depends on the species and the conditions [18]. As an example, although some species belonging to the family Muscidae have been reported from the first species in the sequence of appearance (in buried corpses) [19], they are usually considered late species [12,20].

The genus *Hydrotaea* Robineau–Desvoidy, 1830 (Diptera: Muscidae) includes widely distributed species of Muscidae family, mainly found in warmer regions of the world [19], and its appearance in corpses have been documented in different forensic cases [19,21,22,23,24,25]. Moreover, this genus is considered to belong to a specialized group of flies able to colonize buried remains [19,26,27], an interesting condition in which a lower abundance and diversity of insect species is usually detected. Nonetheless, very little data are available regarding the life cycles of some species of the genus *Hydrotaea*, as well as other aspects of their morphologies, especially those of the preimaginal stages [20,28,29,30,31,32].

In addition, there are almost no studies that consider the biometric parameters of *Hydrotaea* adults, when, for instance, variation in wing morphology has been reported as an important forensic tool to study adults’ development in other Diptera species [33,34,35,36,37].

Within this genus, *Hydrotaea capensis* (Wiedemann, 1818) (Diptera: Muscidae) occurs in most regions of the world, being more abundant in warmer climates, but not in arid ones [30]. It is a synanthropic species, associated with urban or anthropized ecosystems, that has been reported from different forensic contexts all over Europe [19,23,29,38]. It is usually considered a late colonizer and typical of buried corpses. In exposed conditions, *H. capensis* is present during the advanced stages of decomposition, whereas in buried or concealed bodies it tends to be one of the first to colonize [19,39,40,41]. A previous study evaluated the effect of temperature on the development rate of this species but only considered the minimum duration of each stage [29]. This investigation did not undertake a comprehensive study to validate whether morphological characteristics of the immature stages would differ depending on the temperature.

In this context, the present work aimed to study the preimaginal stages development and biometric parameters of adult wings of *H. capensis* to provide information on different parameters of its life cycle, such as the duration of each preimaginal stage, the larval length at each stage and some biometric characteristics of adult wings, depending on temperature. These data should help to establish a better link between the observation of this species and the cases and forensic studies where it is found.

## 2. Materials and Methods

### 2.1. Insect Rearing and Development at Different Temperatures

A *Hydrotaea capensis* colony was established in the laboratory from individuals collected in a forensic case in Murcia (southeast Spain) [21]. The colony was initiated with larvae in the third stage (LIII) and pupae. Larvae were transferred to the Forensic Entomology Laboratory of the Department of Zoology and Physical Anthropology of the University of Murcia. They were kept with pig liver as a food substrate and exposed to a constant temperature of 25 °C, 50% of relative humidity (RH) and a 12:12 photoperiod, in a Sanyo MLR-350H incubator (Sanyo Electric Co. Ltd., Gunma, Japan), until the adults emerged.

To study the life cycle of the species, adults were placed in insectaries (22 × 22 × 25 cm) consisting of a prismatic enclosure on white muslin attached to a frame of wooden rods. Sugar was provided as food for adults, supplied every two days, and water was continuously delivered. Pig liver was used as an egg-laying substrate placed 7 days after the emergence of the adults and kept for 24 h to induce the maturation of the reproductive system in the females [42,43]. Subsequently, the egg-laying substrate was removed from the cages for 48 h to synchronize the female gonadal maturation and the egg laying [15,16]. After this period, the laying substrate was reintroduced into the cage for 2 h. This time interval has been considered adequate in this type of experiment to obtain eggs of the same age (time 0) [44]. The eggs obtained were transferred, together with the egg-laying substrate, and placed in larval cages at 18, 20, 25 and 30 °C, 50–60% RH and 12:12 light:dark photoperiod to complete their development. The larval cages consisted of 20 × 20 × 20 cm containers fitted with a lid to allow access to the interior. The lid had an opening covered with white muslin to allow air circulation inside the cage and to avoid the possible exit of specimens. At the end of the larval phase, the existing pupae were collected and isolated on Petri plates, while waiting for the adults to emerge. Finally, once the adults emerged, they were transferred to the aforementioned insectaries. Four replications were carried out for each temperature.

### 2.2. Sampling and Measurements

The different preimaginal stages were typified as egg, larvae L-I, L-II, L-III and pupal stage. It was considered that the preimaginal population changed from one stage to another when 10% of the individuals studied did so [45,46]. At 18 °C, larvae were collected every 6 h for the first 3 days and every 12 h until stage L-II/L-III was reached. At 20, 25 and 30 °C, samples were taken every 12 h until reaching stage L-III (Appendix A, Table A1). From that moment, and for all temperatures, all the specimens were checked every 24 h until the end of the cycle. The larval specimens were euthanized in boiling water for 30 s, and then preserved in 70% ethanol, according to the protocol developed and standardized by the European Association of Forensic Entomology [5]. The larval length was considered as the distance, in a straight line, between the cephalic lobes, at the anterior end of pseudocephalon, to the posterior spiracles, at the anal division. According to a common practice in forensic entomology, the largest larvae specimens were chosen [47], measuring the length of the larvae in a minimum of five or six individuals up to a maximum of about 10% of the population. The length of each individual was measured using a Leica^®^ MZ8 binocular stereoscope (Leica Microsystems, Heerbrugg, Switzerland) equipped with a micrometric Leica^®^ MOK-95 eyepiece (Leica Microsystems, Heerbrugg, Switzerland), 0.1 mm graduation. For specimens larger than 12 mm, an electronic Vernier caliper (Mitutoyo Absolute Digimatic, CD-10 DCX, Mitutoyo Corporation, Kanagawa, Japan) was used.

### 2.3. Biometric Study

**Figure 1:**
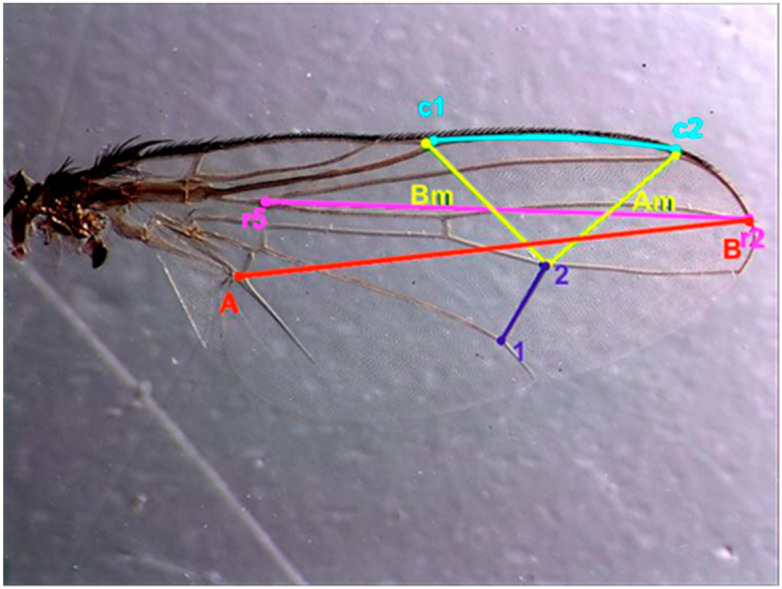
Image of the wing of *Hydrotaea capensis* taken with the binocular stereoscope with the measurements made using the TpsDig2 program according to different morphometric geometry studies. AB, in red; Am/Bm, in yellow; 1–2, in dark blue; c1–c2, in blue; r2–r5, in pink.

The morphometric measurements of the adults at each temperature value were carried out in 15 males and 15 females except for the population bred at 18 °C, in which no adults emerged, and that bred at 20 °C, in which, of all the emerged adults, only three were females. Adults were preserved in glass bottles containing 70% ethanol. The right wing of each individual was removed and placed on a slide with Hoyer’s medium [48]. A camera Leica ® MC170 HD (Leica Microsystems, Heerbrugg, Switzerland) attached to the stereoscope was used to measure it using the free software TpsDig2 (http://life.bio.sunysb.edu/morph/). The following characteristics were measured as an index of adult size (Figure 1, ref. [49]): distance between the vein junctions marked as AB in Figure 1 [35], posterior crossvein (dm–cu) (1–2) [34], costal vein (c1–c2) [37], length of vein R4+5 measured from its junction with r2+3 to the wing apex (r2–r5) [33], the distance between the distal end of the vein R2+3 and the interception point of dm-cu-M1 (Am), and distance between the interception points of dm-cu-M1 and R1-C (Bm) [36].

### 2.4. Data Analysis

A two-way ANOVA was used to analyze the influence of temperature on the larval length in each developmental stage and on the biometric parameters of the wings of adult specimens, using the function “aov” in the “stats” package [50]. To discriminate the average time of development of each preimaginal state and the biometric measurements of the adult wings, for each temperature and between sexes (in the case of the biometric study in adults), an LSD test (α = 0.05) was used with the function “LSD.test” in the “agricolae” package [51]. The different developmental stages were: (0) = egg, (1) = L-I, (2) = L-II, (3) = L-III and (4) = pupal, considering that each stage has passed from one to another when 10% of the individuals studied have done so.

## 3. Results

### 3.1. Development Times

The larval cycle, from laying to the prepupal stage, had a significantly longer duration at 18 °C than the other three temperatures, being the shortest at 30 °C (Table 1). Differences were significant between the average time of larval stages development at all the four temperatures except between 18 °C and 20 °C and between 25 °C and 30 °C in the first two stages. The ranges of minimum and maximum averages of the duration of each stage were between 38.2 ± 1.1 h (at 30 °C) and 111.0 ± 5.1 h (at 18 °C) for L-I, 65.5 ± 1.8 h (at 30 °C) and 194.9 ± 3.3 h (at 20 °C) for L-II, 108.1 ± 1.6 h (at 30 °C) and 367.9 ± 3.2 h (at 18 °C) for L-III, and the pupal stage was reached between 252 ± 27.2 h (at 30 °C) and 498.0 h (at 18 °C), (Table 1, Appendix A Table A1). The first adults emerged after 318 h at 30 °C (13.2 days), and after 378 h (15.7 days) at 25 °C. It is noteworthy that at 18 °C no adults emerged. In addition, at 20 °C, due to problems of accessibility to the laboratory, it was not possible to collect sufficient samples to determine when adults would have emerged and, therefore, to know the duration of the cycle, but it is known that it took more than 470.6 h (more than 19.6 days) at 20 °C.

### 3.2. Changes in the Larval Length over Time and Its Relationship with Temperature

**Figure 2:**
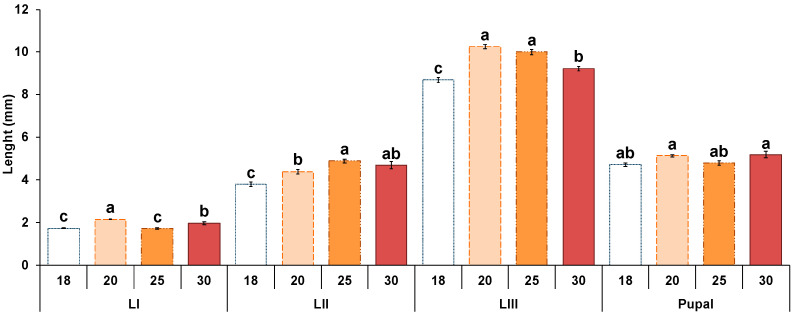
Larval length average in each stage of *Hydrotaea capensis* at 18, 20, 25 and 30 °C. Letters indicate statistically significant differences (LSD test, *p* < 0.05) between temperatures of the same stage.

After hatching, the body length of the larvae progressively increased until they reached the prepupal stage when their size drastically decreased (Appendix A, Table A2 and Figure 2). At 18 °C and 20 °C, the first L-I larvae were observed after 54 h, presenting the smallest maximum lengths recorded (1.32 ± 0.02 mm and 1.3 ± 0.03 mm, respectively). At 18 °C, the maximum length was registered in the L-III stage after 480 h (9.5 ± 0.4 mm) and at 20 °C after 354 h (11.2 ± 0.2 mm). The pupal stage was reached after 498 h at 18 °C and after 474 h at 20 °C. At 25 °C and 30 °C, body length increased faster than at lower temperatures. In both cases, L-I larvae were first recorded after 30 h (1.4 ± 0.02 mm and 1.7 ± 0.06 mm of length, respectively). At 25 °C, the maximum length was registered in both cases in L-III after 174 h (11.4 ± 0.1 mm) and after 138 h at 30 °C (11.1 ± 0.2 mm). The pupal stage was first recorded after 210 h at 25 °C and after 330 h at 30 °C. In all cases, the body length decreased rapidly when the prepupal stage was reached (Appendix A, Table A2).

There were significant differences in each larval stage length with temperature, except in the pupal stage (L-I: F = 40.1, df = 3, *p* < 0.01; L-II: F = 14.7, df = 3, *p* < 0.01; L-III: F = 41.9, df = 3, *p* < 0.01; Pupal: F = 2.1, df = 3, *p* = 0.09) (Figure 2). Finally, when comparing each larval stage length at different temperatures, an increase in size was observed as the temperature raised (Figure 2). However, those differences were not always significant. For instance, in L-I no differences were observed between 18 °C and 25 °C, in the L-II stage between 25 °C and 30 °C and between 20 °C and 30 °C and in the L-III between 20 °C and 25 °C. These data indicate difficulties in extracting a clear pattern regarding the influence of temperature on larval size, other than the smallest size being observed at 18 °C, except in L-I (Appendix A, Table A2, Figure 2).

### 3.3. Biometry of Adult Specimens

There were significant differences between the length of AB, Am, 1-2 and r2-r5 when considering different temperatures (AB: F = 78.1, df = 2, *p* < 0.01; Am: F = 67.3, df = 2, *p* < 0.01; dm-cu 1-2: F = 49, df = 2, *p* < 0.01; r2-r5: F = 81.5; df = 2, *p* < 0.01). For AB, 1–2, Am and r2–r5, the longest length was observed in the specimens bred at 20 °C, both in males and females, and no differences were observed between the length of the specimens bred at 25 °C and 30 °C. However, no differences were observed in the case of Bm length when considering the different temperatures (Table 2). On the other hand, there were significant differences between sexes in Am and Bm lengths (Am: F = 10.9, df = 1, *p* < 0.01; Bm: F = 19.2, df = 1, *p* < 0.01). The Am length was larger in males than in females, with the longest length at 20 °C, while the Bm length was larger in females at all studied temperatures (Table 2). Finally, no differences were observed in the C1–C2 length either when considering the temperature or the sexes.

## 4. Discussion

*Hydrotaea* species have been reported in numerous forensic cases, colonizing bodies in exposed conditions later than Calliphoridae adults showing a clear preference for outdoor crime scenes and being one of the first colonizers in buried or hidden bodies [3,21,22,27,30,39,40,41,52], possibly because of its small size, this makes it easy for it to manoeuvre through interstitial spaces in the ground to reach a carcass [27]. The review by Lutz et al. [3] indicates that the seasonal oviposition activity of this genus on human bodies has two main peaks, in May and August, although it must be considered that their review included cases that occurred in central Europe.

In particular, *H. capensis* is a common species in these forensic scenarios [23,24,25,38], reported in decay/advanced decay stages of body decomposition in exposed conditions, and as one of the first colonizers in buried corpses. Some studies have considered its preimaginal external morphology, such as Turchetto and Vanin [53] and Couri et al. [54,55], who partially describe the third-stage larvae or the puparium, and Paños Nicolás [56] who provides a detailed description of each larval stage of the species, highlighting the most relevant characters that allow their identification. However, very few studies provide data on either their biology or the environmental conditions in which it develops. For instance, Lefebvre and Pasquerault [29] studied the effect of temperature on the developmental rate of this species, but they only considered the minimum duration of each stage of development. Therefore, to date, no comprehensive study has been undertaken to validate whether morphological characteristics of the immature stages and biometrical features of adults of this species would differ depending on a defined environmental variable such as temperature. Our study provides an examination of the development of *H. capensis*, with emphasis on changes in larval length and biometric adult parameters at four different temperatures.

Significant differences were found in the time needed to reach successive larval stages as a function of temperature. Thus, larvae developed at high temperatures (25 °C or 30 °C) and reached the pupal stage much faster than at low temperatures (18 °C or 20 °C). Specifically, in our case, it took an average of 10.5 days (at 30 °C), 13.3 days at 25 °C, 19.7 days at 20 °C and 20.7 at 18 °C to reach the pupal stage. This trend agrees with other studies of different or even the same species of the same genera, *H. rostrata* Robineau-Desvoidy, 1830 [20], *H. capensis* and *H. aenescens* (Wiedemann, 1830) [29] and *H. spinigera* Stein, 1910 [31], and with studies carried out with different species of forensic importance (e.g., *Lucilia sericata* (Meigen, 1826) (Diptera: Calliphoridae) [14], *Protophormia terraenovae* Robineau-Desvoidy, 1830 (Diptera: Calliphoridae) [16] or *Chrysomya albiceps* (Wiedemann, 1819) (Diptera: Calliphoridae) [47], among others). Specifically, Lefebvre and Pasquerault [29] concluded that the duration of the larval development of *H. capensis* could be longer than that of *H. aenescens* and the threshold of development higher than the one of *H. aenescens*. However, this work does not show the complete development of *H. capensis* and, therefore, our data cannot be directly compared with the previous work. Dadour et al. [20] observed that there were large variations in the development rates of *H. rostrata* when exposed to either summer or winter temperature regimes, with a shorter duration in those individuals developed at warmer temperatures. Moreover, the duration of the cycle until pupation in *H. rostrata* at 25 °C was approximately 15 days, a similar result to that reported in our study. Finally, similar results were obtained by Wang et al. [31] working with *H. spinigera* in China, although the duration of the larval cycles was longer in some temperatures than the cycles in our work. Grassberger and Reiter [15] have indicated that, in some cases, differences observed in the duration of larval development could also be influenced by biogeographic factors. In our study, a shortening in the emergence of adults between 25 °C and 30 °C degrees was also observed, but due to the absence of data on emergence at temperatures of 18 °C and 20 °C it is not possible to draw further conclusions.

On the other hand, changes in larval body length are commonly used in forensic entomology as indicators of the development time of a certain species in a corpse [7]. There were significant differences when comparing larval length at different temperatures in our case. However, those differences do not indicate a clear pattern, since the differences between temperatures varied depending on their stage. Furthermore, no differences in length were found between the pupal stages at different temperatures. Pupae are frequently found as entomological evidence in forensic scenarios, but, as other authors have suggested, it is difficult to estimate their age [57], so further studies are needed on this issue using emerging technologies that provide accurate and reliable age estimates [7,23,58,59].

Concerning the biometry of adults, studies of variation in Diptera wing morphology have shown that environmental factors such as climate, temperature, photoperiod, latitude, or larval competition influence wing size [33,34,35,36,37]. Therefore, applying biometric data to the analysis of the wing size of *H. capensis* can provide a new tool for forensic entomology, allowing the study of adult development.

Few studies consider the biometric parameters of *Hydrotaea* adults; therefore, the parameters measured in this work have been selected on the basis that they have been used with other species of flies as indicators of adult sizes [33,34,35,36,37]. For instance, Mullany et al. [37] measured the costal vein in *Calliphora stygia* (Fabricius, 1781) (Diptera: Calliphoridae) to determine if any differences existed between treatments caused by drug exposure during earlier life stages, finding significant differences in the vein size between groups. Saunders and Bee [33] and Smith and Wall [34] considered the r2–r5 vein in *Calliphora vicina* Robineau–Desvoidy, 1830 (Diptera: Calliphoridae) and the posterior crossvein in *C. vicina* and *L. sericata* to compare different fly groups determining that both parameters are useful to compare the size of adult flies. Chapman and Goulson [35] used the AB to observe how the temperature and the duration of larval development affected the asymmetric fluctuations of the wings in *Musca domestica* Linnaeus, 1758 (Diptera: Muscidae). In contrast, in the study by da Silva et al. [36] it was found that the Am and Bm parameters in *Haematobia irritans* (Linnaeus, 1758) (Diptera: Muscidae) did not meet the fluctuating asymmetry criteria, and were therefore omitted from these analyses. All these studies reaffirm the potential of wing morphometry as an indicator of the growth of Diptera under certain environmental conditions, although they also highlight the need to increase the number of studies, under controlled environmental conditions and natural conditions, to strengthen its interpretation.

In our study, significant longest length was found in AB, Am, 1–2 and r2–r5 parameters for specimens bred at 20 °C, but no differences were observed between specimens bred at 25 °C and 30 °C. Moreover, and as suggested by da Silva et al. [36], Am and Bm parameters were not relevant regarding the temperature at which *H. capensis* was developed. Nonetheless, differences were also observed between sex when considering their length. In addition, the length of c1–c2 is revealed as a not relevant parameter for *H. capensis* since it did not present any significant differences in terms of sex or developmental temperature.

## 5. Conclusions

According to different authors such as Charabidze and Martin-Vega [60], the analysis of forensic entomological data together with its environmental variables and the study of the development times of the species and the factors that condition them are issues that should receive more attention that is due to their importance in aspects such as the ageing of the larvae, the growth rates of the entomofauna, and the estimation of the minPMI.

*Hydrotaea capensis* is considered a colonizer that is present during the advanced stages of decomposition in exposed bodies, while in buried or hidden bodies it tends to be one of the first to colonize. Thus, there is a need to have more precise data about its biology to gain insights into the interpretation of its presence in corpses. The relevance of our work is that it is the first study to provide data on the duration of the life cycle of this species, finding significant differences in the average time of development of most larval stages with longer duration at low temperatures and vice versa. However, no clear pattern was found regarding the length of the larvae depending on the temperature beyond the fact that at 18 °C the smallest lengths were recorded.

In the case of adults, it is proposed that some wing biometrical data can be used as indicators of their growth. Wing parameters AB, Am, 1–2 and r2–r5 showed significant differences when considering the different temperatures and sexes in the Am case. However, Bm allows us to differentiate between sexes, but not between the temperatures at which it had developed.

In general, all available data used in forensic practice are mainly focused on the development of early colonizing dipteran species (e.g., *Calliphora* spp., *Lucilia* spp.) [61]. Thus, the data presented in this study will assist forensic entomologists to obtain more accurate minPMI and post-burial interval (PBI) estimations in cases where late colonizer species or buried remains colonizer species, like *H. capensis*, are present.

## Figures and Tables

**Table 1:** Average duration (hours ± standard error, SE) for each preimaginal stage at different rearing temperatures. Different letters indicate statistically significant differences (LSD test, *p* < 0.05).

	STAGES			
Temperature	L-I	L-II	L-III	Pupal
18 °C	111.0 ± 5.1 a	193.5 ± 4.1 a	367.9 ± 3.2 a	498.0 ± 0.0 a
20 °C	107.4 ± 1.0 a	194.9 ± 3.3 a	333.6 ± 2.3 b	470.6 ± 2.4 b
25 °C	45.3 ±1.0 b	73.8 ± 1.2 b	155.6 ± 1.3 c	321.0 ± 3.7 c
30 °C	38.2 ± 1.1 b	65.5 ± 1.8 b	108.1 ± 1.6 d	252.0 ± 27.2 d

**Table 2:** Length (average ± standard error, in mm) of the different biometric wing measurements of *H. capensis* (F: females, M: males) at 20 °C, 25 °C and 30 °C. No adults emerged at 18 °C. Temp. is temperature, N is the sample size. Different letters indicate statistically significant differences (LSD test, *p* < 0.05).

Temp.	Sex	N	A-B	Am	Bm	1-2	C1-C2	r2-r5
20 °C	F	3	3.08 ± 0.02 a	1.06 ± 0.01 a	0.95 ± 0.02 a	0.51 ± 0.01 a	1.47 ± 0.01 a	2.96 ± 0.04 a
	M	15	3.25 ± 0.05 b	1.15 ± 0.02 b	0.78 ± 0.04 b	0.51 ± 0.01 a	1.36 ± 0.08 a	3.04 ± 0.05 a
25 °C	F	15	2.78 ± 0.02 c	0.96 ± 0.01 d	0.88 ± 0.01 a	0.43 ± 0.01 b	1.36 ± 0.02 a	2.60 ± 0.02 b
	M	15	2.82 ± 0.03 c	1.01 ± 0.01 bc	0.81 ± 0.01 b	0.44 ± 0.01 b	1.34 ± 0.01 a	2.63 ± 0.02 b
30 °C	F	15	2.77 ± 0.03 c	0.97 ± 0.01 d	0.88 ± 0.01 a	0.43 ± 0.01 b	1.34 ± 0.02 a	2.59 ± 0.03 b
	M	15	2.75 ± 0.03 c	0.98 ± 0.01 cd	0.81 ± 0.01 b	0.45 ± 0.01 b	1.31 ± 0.02 a	2.56 ± 0.03 b

## Data Availability

Data is contained within the article.

## Appendix A

**Table A1:** Number of specimens of *H. capensis* (N) and stage (L-I, L-II, L-III and pupal) mapped to different sampling intervals (hours) at different constant temperatures.

	18 °C	20 °C	25 °C	30 °C
Time (h)	N	N	N	N
30	-	-	22 LI	36 LI
48	-	-	35 LI	30 LI + 4 LII
54	14 LI	5 LI	28 LI	37 LII
66	-	-	70 LII	-
78	6 LI	-	-	18 LII + 150 LIII
90	-	58 LI	31 LII	11 LII + 35 LIII
114	-	120 LI	1 LII + 26 LIII	37 LIII
126	18 LI +1 LII	24 LI + 17 LII	28 LIII	29 LIII
138	-	1 LI + 38 LII	58 LIII	43 LIII
150	20 LI + 24 LII	31 LII	65 LIII	39 LIII
162	20 LII	31 LII	36 LIII	28 LIII + 6 pupal
174	16 LII	-	37 LIII	-
186	16 LII	-	79 LIII	-
210	-	39 LII + 1 LIII	25 pupal	-
234	17 LII	27 LII + 5 LIII	95 pupal	-
258	14 LII + 1 LIII	42 LII + 19 LIII	-	-
282	4 LII + 10 LIII	14 LII + 85 LIII	67 pupal	-
284	-	-	186 pupal	-
306	2 LII + 15 LIII	42 LIII	-	-
318	-	-	-	*
330	44 LIII	73 LIII	-	3 pupal
354	36 LIII	77 LIII	-	3 pupal
378	48 LIII	67 LIII	5 pupal *	-
402	46 LIII	-	-	-
426	-	36 LIII + 2 pupal	-	-
450	-	-	157 pupal	-
474	-	26 pupal	-	-
480	18 LIII	-	-	-
498	96 pupal	-	-	-

* time from which adults began to emerge.

**Table A2:** Changes in *H. capensis* larval body length (mm) (average ± standard deviation, SD) overtime under different constant temperatures.

Tª	18 °C				20 °C				25 °C				30 °C			
Time	LI	LII	LIII	P	LI	LII	LIII	P	LI	LII	LIII	P	LI	LII	LIII	P
6																
18																
30									1.40 ± 0.02				1.67 ± 0.06			
48									1.71 ± 0.03				2.36 ± 0.05	3.20 ± 0.06		
54	1.32 ± 0.02				1.30 ± 0.03				2.02 ± 0.04					4.62 ± 0.16		
66										4.61 ± 0.09						
78	1.32 ± 0.06													4.11 ± 0.33	7.49 ± 0.07	
90					1.89 ± 0.02					5.51 ± 0.10				6.50 ± 0.46	9.87 ± 0.21	
114					2.28 ± 0.02						7.87 ± 0.27				10.64 ± 0.17	
126	1.97 ± 0.04				2.36 ± 0.04	2.71 ± 0.05					10.32 ± 0.24				11.01 ± 0.18	
138						2.86 ± 0.07					8.96 ± 0.30				11.13 ± 0.19	
150	1.97 ± 0.05	2.61 ± 0.07				3.29 ± 0.10					9.52 ± 0.24				10.67 ± 0.23	
162		3.00 ± 0.14				3.97 ± 0.13					9.93 ± 0.31				9.14 ± 0.27	5.20 ± 0.31
174		3.16 ± 0.17									11.39 ± 0.14					
186		3.74 ± 0.21									11.16 ± 0.15					
210						5.41 ± 0.10						5.04 ± 0.04				
234		5.11 ± 0.22				5.49 ± 0.24	7.62 ± 0.23					3.91 ± 0.13				
258		5.42 ± 0.18				5.50 ± 0.16	8.37 ± 0.28									
282		5.65 ± 0.28	8.14 ± 0.54			5.74 ± 0.22	9.20 ± 0.16					4.83 ± 0.10				
284												5.03 ± 0.05				
306		5.55 ± 0.55	7.60 ± 0.37				10.23 ± 0.24									
330			9.07 ± 0.25				10.59 ± 0.21									5.27 ± 0.09
354			9.31 ± 0.29				11.18 ± 0.18									5.07 ± 0.09
378			8.46 ± 0.28				10.88 ± 0.26					5.08 ± 0.10				
402			8.28 ± 0.30													
426							10.43 ± 0.23	5.30 ± 0.30								
450												4.97 ± 0.07				
474								5.12 ± 0.04								
480			9.51 ± 0.44													
498				4.72 ± 0.09												
